# Impact of Periodontal Diseases on Oral Health-Related Quality of Life: A Study with a Condition-Specific Questionnaire in Croatian Population

**DOI:** 10.1055/s-0044-1785534

**Published:** 2024-08-05

**Authors:** Aleksandar Pupovac, Davor Kuiš, Ivana Mišković, Jelena Prpić

**Affiliations:** 1 Department of Periodontology, Faculty of Dental Medicine, University of Rijeka, Rijeka, Croatia; 2 Department of Periodontology, Clinic of Dental Medicine, Clinical Hospital Center Rijeka, Rijeka, Croatia; 3 Department of Dental Medicine, Faculty of Dental Medicine and Health, University of Osijek, Osijek, Croatia

**Keywords:** oral health, periodontal diseases, quality of life, smoking, validation

## Abstract

**Objectives** The aim of this study was to translate and validate the condition-specific Oral Health Impact Profile (OHIP) in the Croatian cultural context and assess the impact of periodontal diseases and nonsurgical periodontal therapy on quality of life (QoL).

**Materials and Methods** A cross-sectional study was carried out on 150 individuals: 50 periodontally healthy, 50 with gingivitis, and 50 with periodontitis who self-administrated the OHIP. The participants' age ranged between 18 and 71 years, with the median age of 45 (34–57) years. Forty-seven percent of the participants were females. The validity and reliability of the Croatian OHIP version were tested. The impact of gingivitis and periodontitis on QoL was assessed. Changes in QoL induced by nonsurgical periodontal therapy in 20 patients with periodontitis were analyzed.

**Statistical Analysis** Categorical data were presented by absolute and relative frequencies. The normality of the distribution of continuous variables was tested by the Shapiro–Wilk test. Continuous data were described by the median and the limits of the interquartile range (IQR). Differences in continuous variables between two independent groups were tested with the Mann–Whitney *U* test, and between three groups with the Kruskal–Wallis test (post hoc Conover). The Wilcoxon signed-rank test was used to examine the differences in the total score before and after therapy. All *p* values were two-sided. The level of significance was set at alpha of 0.05.

**Results** The analysis detected a single-factor structure that explained for the 56.9% of the variance. Cronbach's alpha value was 0.937, which indicated an excellent internal consistency. Overall OHIP score reported a strong correlation with the subjective estimate of periodontal problems (Rho = 0.92; *p* < 0.001). Test–retest reliability was high ( *r* = 0.984; *p* < 0.001). The periodontitis group had the highest OHIP score (28 [23–34]), followed by the gingivitis group (14 [12–20]) and the periodontally healthy group (9 [5–11]; *p* < 0.001). Nonsurgical periodontal therapy significantly improved the QoL in those with periodontitis ( *p* < 0.001).

**Conclusion** The condition-specific Croatian version of the OHIP instrument can be considered adequate to measure the impact of periodontal diseases on oral health–related QoL. Periodontal diseases, especially periodontitis, have a negative effect on the patient's QoL. Nonsurgical periodontal treatment can improve patients' QoL.

## Introduction

The World Health Organization defines quality of life (QoL) as people's perception of their position in life in the context of culture and value systems in which they live and in relation to their goals, expectations, standards, and concerns. 1 Oral health is an important part of an individual's well-being, and oral conditions can alter one's QoL. Oral health is assessed by various clinical measures that help evaluate the presence or progression of a disease. Despite their importance, our focus should also be on inclusion of the patient's perception of the disease in order to provide a comprehensive assessment of their condition and treatment needs.

Oral health–related quality of life (OHRQoL) measurement is quite challenging as it is subjective and difficult to interpret and draw a valid conclusion for different cultural contexts. It is measured by various types of questionnaires that assess the impact of oral conditions and diseases on the patient's life. Most questionnaires concentrate on functional, social, and psychological impacts. One of the most commonly used instruments to measure OHRQoL is the Oral Health Impact Profile (OHIP). It was developed by Slade and Spencer in 1994 and consists of 49 items that cover 7 domains. 2 In 1997, Slade developed a shorter 14-item form, the OHIP-14. 3 The instrument was validated and culturally adapted in different populations and translated to many languages. OHIP-14 is a generic questionnaire applicable to a great number of oral conditions and diseases, including periodontal diseases. Periodontal diseases cause many symptoms such as bleeding, swollen gums, pain, halitosis, and tooth mobility, with periodontitis being a major cause of tooth loss. The disease has a negative impact on a patient's function, comfort, appearance, and self-confidence. 4 Three systematic reviews from 2016, 2017, and 2020 concluded that OHRQoL is affected by periodontal diseases, especially by severe stages of periodontitis. 5 6 7 Due to their specificity, some researchers suggested developing and using a condition-specific questionnaire in further research on the impact of periodontal diseases on patients' QoL. 7 8 9 Two different condition-specific questionnaires have been developed in 2017: the Oral Health Impact Profile-14 applied to Periodontal Diseases (OHIP-14-PD) by Moral De la Rubia and Franco 10 and the Oral Health Impact Profile for Chronic Periodontitis (OHIP-CP) by He et al. 9 OHRQoL measures are suggested to be implemented in new studies' design to complement clinical data. 8 11 Upon analyzing the conclusions of the earlier-mentioned systematic reviews, it can be observed that they generally fail to use the condition-specific instruments in assessing OHRQoL and that there are very few studies with such questionnaires so far. Therefore, the aim of this study was to translate and validate the OHIP-14-PD in the Croatian cultural context and assess the impact of periodontal diseases and nonsurgical therapy on QoL.

## Materials and Methods

### Oral Health Impact Profile Applied to Periodontal Diseases

The OHIP-14-PD developed by Moral De la Rubia and Franco was used to measure the impact of periodontal diseases on patients' QoL. 10 The OHIP-14-PD questionnaire measures QoL using 14 items. The use of OHIP-14-PD is recommended as a one-dimensional measure. 10 Each item has a set of five possible answers based on a Likert scale (0 = never; 1 = almost never, 2 = occasionally, 3 = frequently; 4 = very frequently) which describe how frequently the participants experienced a negative impact of various periodontal conditions on their well-being and QoL in the past 6 months. The overall score is calculated by summing all items and ranges from 0 to 56 points.

The OHIP-14-PD questionnaire was originally developed in English and Spanish, 10 so it had to be translated to Croatian and adapted to the Croatian cultural setting and context. It was translated and adapted using the back-translation technique. 12 The English version was independently translated to Croatian by two dentists whose native language is Croatian, both fluent in English and one of them specialized in periodontology and familiar with the OHRQoL instruments. Following that, a study group of five members (2 periodontists, 2 periodontology residents, and 1 oral surgeon), all fluent in English, reviewed and compared the two drafts and produced a consensus draft version. This version was back-translated to the English language by a native professional translator fluent in both languages and unfamiliar with the original version. The same study group reviewed again the items of the forward and backward translations and compared them to the original version to check for possible inconsistencies. A final Croatian version (OHIP-14-PD-CRO; Table 1 ) was created and approved by the authors and the study group. A pilot study was caried out on a convenient sample of 20 participants. It was aimed to verify the semantics, syntax, clarity, and an overall understanding of the items in the questionnaire. Some minor linguistic changes were made accordingly.

**Table 1:** OHIP-14-PD and OHIP-14-PD-CRO

Have you noticed your gums are swollen and do not look good?	Jeste li primijetili da je Vaše zubno meso natečeno i ne izgleda dobro?	0	1	2	3	4
Have you had difficulty chewing because of mobility and change of position of your teeth?	Jeste li imali poteškoća sa žvakanjem zbog pomičnosti i promjene položaja Vaših zuba?	0	1	2	3	4
Have you felt pain in your gums?	Jeste li osjećali bol u zubnom mesu?	0	1	2	3	4
Have you had sensitive teeth when chewing due to cold, hot, or sweet foods or drinks?	Jesu li Vam zubi bili osjetljivi prilikom žvakanja ili prilikom konzumacije hladne/tople/slatke hrane ili pića?	0	1	2	3	4
Have you been worried because of bad taste in your mouth?	Jeste li bili zabrinuti zbog lošeg okusa u Vašim ustima?	0	1	2	3	4
Have you felt uncomfortable because of bad mouth odor?	Jeste li se osjećali nelagodno zbog lošeg zadaha?	0	1	2	3	4
Has your oral hygiene been inadequate because of gum bleeding when brushing?	Je li Vaša oralna higijena bila neodgovarajuća zbog krvarenja zubnog mesa tijekom četkanja?	0	1	2	3	4
Have you avoided chewing with all your teeth because of any absence of dental pieces or accumulation and/or food residue between the teeth?	Jeste li izbjegavali žvakati s pojedinim zubima zbog nedostatka dijelova zuba ili zaostajanja hrane između zuba?	0	1	2	3	4
Have you felt sad about the health condition of your teeth and gums?	Jeste li se osjećali tužno zbog stanja Vaših zuba i zubnog mesa?	0	1	2	3	4
Have you felt embarrassed by the appearance of your teeth and gums?	Jeste li se osjećali neugodno zbog izgleda Vaših zuba i zubnog mesa?	0	1	2	3	4
Have you had difficulty doing any daily activities because of the state of your teeth or your gum disease?	Jeste li imali poteškoća u obavljanju nekih svakodnevnih aktivnosti zbog stanja Vaših zuba ili bolesti zubnog mesa?	0	1	2	3	4
Have you avoided any contact with other people because of the state of your teeth or your gum disease?	Jeste li izbjegavali kontakt s drugim ljudima zbog stanja Vaših zuba ili bolesti zubnog mesa?	0	1	2	3	4
Has your general health been affected as a result of your oral health?	Je li na Vaše opće zdravlje utjecalo zdravlje Vaše usne šupljine?	0	1	2	3	4
Has your financial situation been affected by the state of your oral health?	Je li na Vašu financijsku situaciju utjecalo zdravstveno stanje Vaše usne šupljine?	0	1	2	3	4

Abbreviations: OHIP-14-PD, the Oral Health Impact Profile- 14 applied to Periodontal Diseases; OHIP-14-PD-CRO, the Oral Health Impact profile-14 applied to Periodontal Diseases Croatian version.

Subsequently, the psychometric properties, validity, and reliability of the Croatian version were tested. The face and content validity have already been assessed during the pilot study. The structural validity of the questionnaire was checked by an explanatory factor analysis. Convergent validity was tested with the Spearman correlation between OHIP-14-PD-CRO score and overall score of another scale with similar construct measuring a subjective estimation of periodontal problems (bleeding gums, swollen gums, calculus, tooth mobility). Discriminative validity was assessed by comparing the scores of the participants by sex, tobacco smoking, and periodontal status. A group of 30 participants was asked to complete the questionnaire 2 weeks apart without any dental intervention to assess the test–retest reliability calculating the intraclass correlation coefficients (ICC). Internal consistency was determined by calculating Cronbach's alpha and alpha if an item was deleted. Values greater than 0.75 were considered good, and values greater than 0.90 indicated excellent reliability. 13 14 OHIP-14-PD-CRO responsiveness was tested on 20 participants before and after nonsurgical periodontal treatment.

### Participants and Data Collection

The study was performed in accordance with the principles outlined in the Helsinki Declaration and was approved by the Ethics Committee of the Faculty of Medicine of the University of Rijeka. All the participants involved in the study were thoroughly informed about it and they gave a written consent.

This cross-sectional study was carried out at the Clinic of Dental Medicine, Clinical Hospital Center Rijeka, Croatia and at one author's (AP) private dental practice during the year 2023. A total of 150 participants were recruited among patients seeking dental treatment or regular checkups. A stratified sampling method was used during the recruitment. The patients were clinically examined and divided into three groups based on their periodontal status. The first group consisted of periodontally healthy patients; the second group included patients with gingivitis; and the third group comprised patients with periodontitis. Periodontal health, gingivitis, and periodontitis were diagnosed according to the criteria outlined in the 2017 Classification of Periodontal and Peri-Implant Diseases and Conditions. 15 16 17 18 All the participants were asked to anonymously self-administrate the OHIP-14-PD-CRO questionnaire. For test–retest, a convenient sample of 30 participants was selected from among students and acquaintances. For responsiveness testing, 20 patients from the “periodontitis” group filled out the questionnaire 6 weeks after nonsurgical periodontal therapy. The exclusion criteria were the following: minors, lack of consent, inability to read and understand the questions, presence of removable dentures and acute symptomatic oral problems. Any patient who met the mentioned criteria was excluded and replaced by the next patient.

### Statistical Analysis

Categorical data were presented by absolute and relative frequencies. The normality of the distribution of continuous variables was tested by the Shapiro–Wilk test. Continuous data were described by the median and the limits of the interquartile range (IQR). Differences in continuous variables between two independent groups were tested with the Mann–Whitney *U* test, and between three groups with the Kruskal–Wallis test (post hoc Conover). The Wilcoxon signed-rank test was used to examine differences in the total score before and after therapy. All *p* values were two-sided. The level of significance was set at alpha of 0.05. The statistical analysis was performed using MedCalc Statistical Software version 22.006 (MedCalc Software Ltd, Ostend, Belgium; https://www.medcalc.org ; 2023) and the IBM SPSS Stat. 23 (IBM Corp. Released 2015. IBM SPSS Statistics for Windows, Version 23.0. Armonk, NY, United States).

## Results

### Participant's Characteristics

A total of 150 patients participated in the study. Fifty patients were periodontally healthy, 50 had gingivitis, and 50 had periodontitis. Seventy-nine patients (53%) were males and 71 (47%) were females. The participants' age ranged between 18 and 65 years, with the median age of 43 (31–57) years. Forty-six subjects (31%) were tobacco smokers. In the sample for test–retest, the median age was 25 (24–33) years and 60% were females. In the sample used for responsiveness testing, the median age was 45 (37–58) years and 55% were females.

### Psychometric Properties of OHIP-14-PD-CRO

**Fig. 1:**
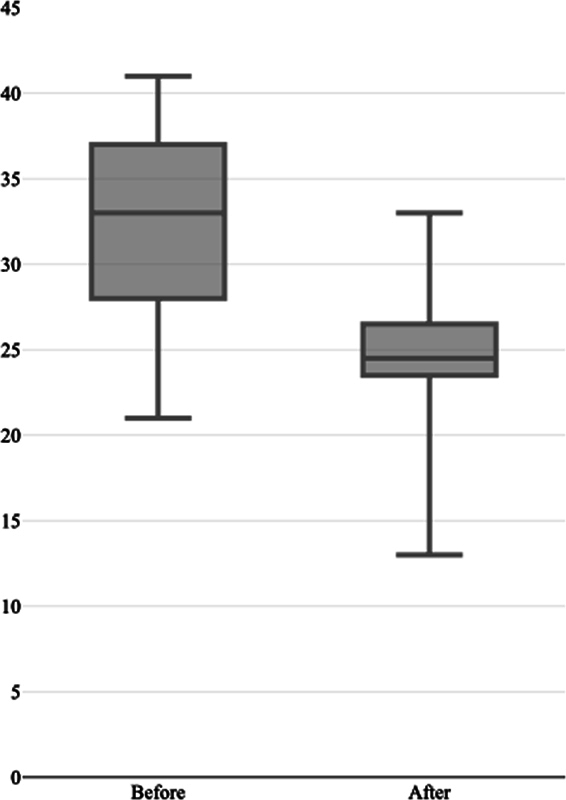
Overall scores before and after nonsurgical periodontal therapy.

Face and content validity was confirmed during the pilot testing with no major changes to the items. Structural validity was assessed by explanatory factor analysis using the Guttman–Kaiser criterion and scree plot. The analysis detected a single-factor structure, which explained for the 56.9% of the variance. A single-factor structure was retained, as in the original version of the questionnaire. Convergent validity was tested, and the OHIP-14-PD-CRO scores reported a strong correlation with the subjective estimate of periodontal problems with the Spearman correlation Rho value of 0.92 ( *p* < 0.001). Discriminative validity testing showed that there were no significant differences in scoring by subjects compared by sex and tobacco smoking in the “healthy” and “periodontitis” groups, while in the “gingivitis” group, nonsmokers and females reported higher frequencies of complains. The instrument demonstrated the ability to differentiate between the three groups (healthy, gingivitis, periodontitis). Test–retest reliability was high with an ICC value of 0.984 (95% confidence interval [CI]: 0.967–0.993; *p* < 0.001). Cronbach's alpha value was 0.937 ( *p* < 0.001), which indicated an excellent internal consistency. Cronbach's alpha value was not increased by deleting any item with values ranging between 0.926 and 0.939, so all the original items were included in the final version. Responsiveness tests showed that the questionnaire was able to detect differences in the subject's QoL before and after nonsurgical periodontal therapy. The overall score before therapy was higher than that after therapy (Wilcoxon test; *p* < 0.001). Results are presented in Fig. 1 .

### Impact of Periodontal Diseases on QoL

**Fig. 2:**
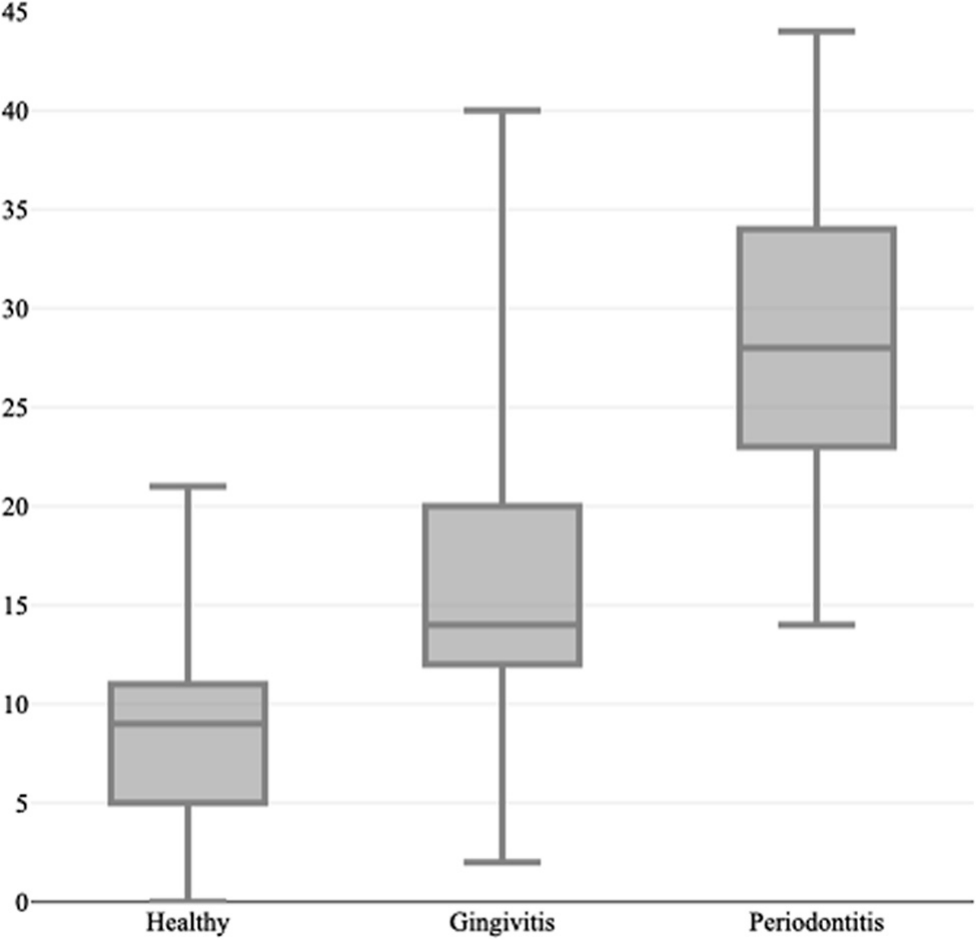
Comparison of overall scores between the three groups.

The distribution of responses to each OHIP-14-PD-CRO item and overall scores in each group are presented in Table 2 . The lowest overall score was in the periodontally healthy group with an average score of 9 (5–11). Both groups with periodontal disease showed higher overall scores. Patients with gingivitis had an average score of 14 (12–20), followed by a significantly higher score of 28 (23–34) in the group with periodontitis ( *p* < 0.001; Fig. 2 ). In the healthy group and in the group with periodontitis, there were no differences in the QoL between male and female patients nor between tobacco smokers and nonsmokers. In the group with gingivitis, females felt more uncomfortable about bad breath than males ( *p* = 0.03). In the same group, nonsmokers declared that their QoL was more affected by problems with dental hypersensitivity ( *p* = 0.03), were more concerned about bad taste and breath ( *p* = 0.04 and 0.03, respectively), and missing dental elements ( *p* = 0.03). Moreover, they reported being sadder and more embarrassed because of their teeth or gums ( *p* = 0.01 in both) and they were spending more money on their oral health compared to smokers ( *p* = 0.02).

**Table 2:** Distribution of responses in the three groups

	Median (IQR)		
	Healthy (H)	Gingivitis (G)	Periodontitis (P)
Item 1	1 (0–1)	2 (1–2)	3 (2–3)
Item 2	0 (0–0)	0 (0–1)	2 (1–3)
Item 3	1 (1–1)	1 (1–2)	2 (2–3)
Item 4	1 (1–2)	2 (1–2)	2 (2–3)
Item 5	1 (0–1)	1 (1–1)	2 (2–2)
Item 6	1 (0–1)	1 (1–2)	2 (2–3)
Item 7	1 (1–1)	2 (1–2)	2 (2–3)
Item 8	1 (0–1)	1 (0–1,25)	2 (2–3)
Item 9	0 (0–1)	1 (1–2)	2 (1–3)
Item 10	0 (0–1)	1 (1–2)	2 (2–2)
Item 11	0 (0–0)	0 (0–0)	1 (0,75–2)
Item 12	0 (0–0)	0 (0–1)	1 (1–2)
Item 13	0 (0–0)	0 (0–0)	1 (1–1)
Item 14	1 (1–2)	2 (1–2)	2 (2–3)
Overall score a b	9 (5-11)	14 (12-20)	28 (23-34)

Abbreviation: IQR, interquartile range.

a Kruskal–Wallis test (post hoc Conover test; H/G: *p* < 0.001; H/P: *p* < 0.001; G/P: *p* < 0.001).

b *α* = 0.05.

## Discussion

In this study, results obtained by measuring the OHRQoL with a condition-specific instrument adapted to the Croatian cultural context showed that periodontal diseases have an impact on patients' QoL and that nonsurgical periodontal therapy is actually able to improve it. In the last few years, there has been a shift from traditional clinically based research to more patient-oriented research, with the patient's well-being and QoL in the focus. A systematic review by Orlandi et al 11 suggested that QoL and patient-based outcomes should be included in future clinical trials and researches. Moreover, an umbrella review of systematic reviews regarding periodontal diseases and QoL concluded that periodontal diseases have a negative impact on QoL and that periodontal treatment can improve patients' QoL. 7 OHIP-14 is one of the most commonly used instruments for the evaluation of the effect of oral diseases and conditions on QoL. It is without any doubt practical to use and widely researched; however, its generalized items also pose as its main flaw as they do not take into consideration specific symptoms of periodontal diseases and the way they affect QoL. Many authors suggested that a condition-specific instrument should be developed and validated for assessment of the impact of periodontal diseases on QoL to overcome this drawback. 5 7 8 19 In 2017, two different groups of authors developed instruments specific for periodontal diseases. He et al 9 created the OHIP questionnaire for chronic periodontitis and Moral De la Rubia and Franco 10 developed the OHIP-14 applied to periodontal disease. To use the latter on a Croatian population, it had to be translated, culturally adapted, and validated. Questionnaires without adequate psychometric properties should be avoided in research. 20 21 To the best of our knowledge, this is the first validation of an instrument specific for periodontal diseases in our country, the first study on the impact of periodontal diseases on QoL in a Croatian population, and one of the very few existing studies with a periodontal-specific questionnaire in general. Many previous studies used various types of generic questionnaires leading to very heterogenous results, which are difficult to compare, interpret, and draw valid general conclusions from.

The explanatory factor analysis of OHIP-14-PD-CRO confirmed the single-factor structure of the original version, which differed from the three-factorial structure of OHIP-CP and the seven-factor structure of OHIP-14. Reliability was evaluated by testing internal consistency and test–retest reliability. Cronbach's alpha was 0.937. This high alpha value demonstrates high correlation between the items. The original OHIP-14-PD and OHIP-CP reported similar alpha values, 0.928 and 0.936. All three questionnaires reported slightly higher alpha values compared to various versions of OHIP-14, ranging from 0.85 to 0.93. 22 The ICC value of 0.984 showed excellent agreement, with similar or higher values compared to other questionnaires. 9 10 23 However, because of the different methodologies and instruments, it is difficult to compare the results between studies.

OHIP-14-PD-CRO demonstrated good discriminant validity and was able to differentiate the “healthy,” “gingivitis,” and “periodontitis” groups with significant differences between total score means, which is in accordance with other validation studies. 9 10 23 In this study, a population stratified by periodontal status was used intentionally to compare the QoL in the three “main” periodontal diagnoses. Many other studies used convenient samples of general population or focused on patients with periodontitis, and most systematic reviews targeted studies with patients with periodontal diseases (both periodontitis and gingivitis) or periodontitis. In addition, the few studies with periodontal-specific OHIP used patients with periodontal diseases or periodontitis only, with no control group of periodontally healthy individuals. Comparing our results with the results by Moral De la Rubia and Franco, 10 the mean value of the overall score in our sample was slightly lower than that in the Mexican population. In the gingivitis group, similar results were obtained. In the Mexican population, the mean score of the patients with gingivitis was equivalent to the one in the general population of adolescents. Meanwhile, in the Croatian population we could differentiate the periodontally healthy patients from patients with gingivitis, with the former having lower scores, suggesting gingivitis also has a negative effect on QoL. These findings may be explained by the cultural differences. In both studies, patients with periodontitis had the highest scores, with the Croatian group having higher results. Regarding smoking, we found no differences between smokers and nonsmokers in the healthy group and in the group with periodontitis. These results are in contrast with various studies that found that smokers had poorer OHRQoL. 24 25 26 This might be explained by the use of a condition-specific questionnaire and focus on a population with periodontal problems. Patients with periodontitis having more problems (e.g., pain, discomfort) associated the bad impact on their QoL more with the disease as such than with smoking. On the other hand, healthy patients having less or no problems did not associate smoking with their OHRQoL. Furthermore, in the previously mentioned studies, samples with general oral problems were surveyed with a more general questionnaire—the OHIP-14. In the gingivitis group, nonsmokers were sadder/more embarrassed with their teeth and gums and declared that dental hypersensitivity, bad taste and breath, and expenses related to oral problems affected their QoL. This might suggest that in this transitional phase from periodontal health to periodontitis, nonsmokers are more concerned about their oral health and their health in general. Periodontitis causes more disability, handicap, and discomfort and has a greater impact on patients' QoL than gingivitis. The findings in our study, although obtained with a different questionnaire, confirmed the findings and conclusions by multiple studies and systematic reviews. 5 6 7 9 10 27

A systematic review from 2013 by Shanbhag et al 19 suggested that all forms of nonsurgical periodontal therapy can improve the OHRQoL of adult patients with periodontal disease in the short and long term. A more recent systematic review from 2021 reported a similar conclusion. 28 The results obtained in this study show that nonsurgical periodontal therapy has a beneficial impact on patients' QoL, which is in accordance with the conclusions of the mentioned reviews. Several studies reported the before and after nonsurgical therapy overall OHRQoL scores 29 30 31 ; however, a direct comparison of the scores should be avoided as different QoL instruments were used. Such problems could be avoided by replacing the generic instruments with a specific instrument. Moreover, a simple comparison of the before and after treatment scores might show paradoxical findings due to the influence of some nontreatment factors on the patient's QoL. 28

The main strengths of our study are certainly the use of an OHIP questionnaire applied to periodontal diseases for QoL measurement as well as a stratified population that comprised individuals with different periodontal status (periodontal health, gingivitis, periodontitis), which enabled us to analyze as objectively as possible the connection between periodontal status and its impact on patients' QoL. Further research with such instruments on a larger number of patients and in different populations is recommended to obtain comparable results between populations as well as to compare data with already existing data gathered with OHIP-14. Moreover, studies on a population with periodontitis stratified by stage and grade with a periodontal-specific OHIP are suggested. Condition-specific questionnaires should be preferred over generic ones in future research and patient-based outcomes should be included in new study designs.

## Conclusion

The condition-specific Croatian version of the OHIP-14 instrument can be considered adequate to measure the impact of periodontal diseases on OHRQoL. Periodontal diseases, especially periodontitis, have a negative effect on the patient's QoL. Nonsurgical periodontal treatment can improve a patient's QoL. Further research with questionnaires adapted for periodontal diseases is recommended.

## References

[1] The World Health Organization Quality of Life assessment (WHOQOL): position paper from the World Health Organization Soc Sci Med 1995 41 10 1403 1409 8560308 10.1016/0277-9536(95)00112-k

[2] Slade G D Spencer A J Social impact of oral conditions among older adults Aust Dent J 1994 39 06 358 364 7832683 10.1111/j.1834-7819.1994.tb03106.x

[3] Slade G D Derivation and validation of a short-form Oral Health Impact Profile Community Dent Oral Epidemiol 1997 25 04 284 290 9332805 10.1111/j.1600-0528.1997.tb00941.x

[4] Needleman I McGrath C Floyd P Biddle A Impact of oral health on the life quality of periodontal patients J Clin Periodontol 2004 31 06 454 457 15142215 10.1111/j.1600-051X.2004.00498.x

[5] Buset S L Walter C Friedmann A Weiger R Borgnakke W S Zitzmann N U Are periodontal diseases really silent? A systematic review of their effect on quality of life J Clin Periodontol 2016 43 04 333 344 26810308 10.1111/jcpe.12517

[6] Ferreira M C Dias-Pereira A C Branco-de-Almeida L S Martins C C Paiva S M Impact of periodontal disease on quality of life: a systematic review J Periodontal Res 2017 52 04 651 665 28177120 10.1111/jre.12436

[7] Wong L B Yap A U Allen P F Periodontal disease and quality of life: umbrella review of systematic reviews J Periodontal Res 2021 56 01 1 17 32965050 10.1111/jre.12805

[8] Graziani F Tsakos G Patient-based outcomes and quality of life Periodontol 2000 2020 83 01 277 294 32385874 10.1111/prd.12305

[9] He S Wang J Wei S Ji P Development and validation of a condition-specific measure for chronic periodontitis: Oral Health Impact Profile for chronic periodontitis J Clin Periodontol 2017 44 06 591 600 28278366 10.1111/jcpe.12716

[10] Moral de la Rubia J Franco N Validation of the Oral Health Impact Profile applied to patients with periodontal disease Rev Fac Odontol Univ Nac (Cordoba) 2017 29 148 172

[11] Orlandi M Muñoz Aguilera E Marletta D Petrie A Suvan J D'Aiuto F Impact of the treatment of periodontitis on systemic health and quality of life: a systematic review J Clin Periodontol 2022 49 24 314 327 34791686 10.1111/jcpe.13554

[12] Brislin R W Back-translation for cross-cultural research J Cross Cult Psychol 1970 1 03 185 216

[13] Bland J M Altman D G Cronbach's alpha BMJ 1997 314 7080 572 9055718 10.1136/bmj.314.7080.572 PMC2126061

[14] Terwee C B Bot S D de Boer M R Quality criteria were proposed for measurement properties of health status questionnaires J Clin Epidemiol 2007 60 01 34 42 17161752 10.1016/j.jclinepi.2006.03.012

[15] Caton J G Armitage G Berglundh T A new classification scheme for periodontal and peri-implant diseases and conditions: introduction and key changes from the 1999 classification J Periodontol 2018 89 01 S1 S8 29926946 10.1002/JPER.18-0157

[16] Lang N P Bartold P M Periodontal health J Periodontol 2018 89 01 S9 S16 29926938 10.1002/JPER.16-0517

[17] Trombelli L Farina R Silva C O Tatakis D N Plaque-induced gingivitis: case definition and diagnostic considerations J Periodontol 2018 89 01 S46 S73 29926936 10.1002/JPER.17-0576

[18] Papapanou P N Sanz M Buduneli N Periodontitis: consensus report of workgroup 2 of the 2017 World Workshop on the Classification of Periodontal and Peri-Implant Diseases and Conditions J Periodontol 2018 89 01 S173 S182 29926951 10.1002/JPER.17-0721

[19] Shanbhag S Dahiya M Croucher R The impact of periodontal therapy on oral health-related quality of life in adults: a systematic review J Clin Periodontol 2012 39 08 725 735 22694297 10.1111/j.1600-051X.2012.01910.x

[20] Persic S Milardovic S Mehulic K Celebic A Psychometric properties of the Croatian version of the Orofacial Esthetic Scale and suggestions for modification Int J Prosthodont 2011 24 06 523 533 22146251

[21] Peršić S Palac A Bunjevac T Celebić A Development of a new chewing function questionnaire for assessment of a self-perceived chewing function Community Dent Oral Epidemiol 2013 41 06 565 573 23551089 10.1111/cdoe.12048

[22] Navabi N Nakhaee N Mirzadeh A Validation of a Persian version of the Oral Health Impact Profile (OHIP-14) Iran J Public Health 2010 39 04 135 139 23113047 PMC3481693

[23] Zasčiurinskienė E Šidlauskas A Kavaliauskienė A Vazgytė J Matuzas A Zaborskis A Reliability and validity of a Lithuanian version of the Oral Health Impact Profile: a study in patients with stage III–IV periodontitis Medicina (Kaunas) 2022 59 01 69 36676693 10.3390/medicina59010069 PMC9867273

[24] Sagtani R A Thapa S Sagtani A Smoking, general and oral health related quality of life: a comparative study from Nepal Health Qual Life Outcomes 2020 18 01 257 32736560 10.1186/s12955-020-01512-y PMC7395342

[25] Thai Cohort Study Team Yiengprugsawan V Somkotra T Seubsman S A Sleigh A C Oral health-related quality of life among a large national cohort of 87,134 Thai adults Health Qual Life Outcomes 2011 9 42 21668968 10.1186/1477-7525-9-42 PMC3125311

[26] Caglayan F Altun O Miloglu O Kaya M D Yilmaz A B Correlation between oral health-related quality of life (OHQoL) and oral disorders in a Turkish patient population Med Oral Patol Oral Cir Bucal 2009 14 11 e573 e578 19680207 10.4317/medoral.14.e573

[27] Al Habashneh R Khader Y S Salameh S Use of the Arabic version of Oral Health Impact Profile-14 to evaluate the impact of periodontal disease on oral health-related quality of life among Jordanian adults J Oral Sci 2012 54 01 113 120 22466895 10.2334/josnusd.54.113

[28] Khan S Khalid T Bettiol S Crocombe L A Non-surgical periodontal therapy effectively improves patient-reported outcomes: a systematic review Int J Dent Hyg 2021 19 01 18 28 32594621 10.1111/idh.12450

[29] Theodoridis C Violesti A Nikiforidou M Menexes G C Vouros I D Short-term impact of non-surgical and surgical periodontal therapy on oral health-related quality of life in a Greek population: a prospective cohort study Dent J 2020 8 02 54 10.3390/dj8020054 PMC7344508 32466149

[30] Vivek B Ramesh K SV Gautami P S Sruthima G NVS Dwarakanath C Anudeep M Effect of periodontal treatment on oral health-related quality of life: a randomised controlled trial J Taibah Univ Med Sci 2021 16 06 856 863 34899130 10.1016/j.jtumed.2021.07.002 PMC8626792

[31] Wong R M Ng S K Corbet E F Keung Leung W Non-surgical periodontal therapy improves oral health-related quality of life J Clin Periodontol 2012 39 01 53 61 22092418 10.1111/j.1600-051X.2011.01797.x

